# Co-Occurrence of Hypophysitis and Thyroiditis Induced by PD-L1 Inhibitor Avelumab: Clinical Insights

**DOI:** 10.1210/jcemcr/luaf093

**Published:** 2025-05-12

**Authors:** Zhanna Zavgorodneva, Muzammil Khan, Helena Guber

**Affiliations:** Division of Endocrinology, Diabetes & Metabolism, Veterans Affairs New York Harbor Healthcare System, Brooklyn, NY 11209, USA; Division of Endocrinology, Diabetes & Metabolism, State University of New York, Downstate Medical Center, Brooklyn, NY 11203, USA; Division of Endocrinology, Diabetes & Metabolism, Veterans Affairs New York Harbor Healthcare System, Brooklyn, NY 11209, USA; Division of Endocrinology, Diabetes & Metabolism, State University of New York, Downstate Medical Center, Brooklyn, NY 11203, USA; Division of Endocrinology, Diabetes & Metabolism, Veterans Affairs New York Harbor Healthcare System, Brooklyn, NY 11209, USA

**Keywords:** immune checkpoint inhibitors, endocrinopathy, hypophysitis, thyroiditis

## Abstract

Various endocrinopathies have been described in patients with malignancies who are receiving treatment with immune checkpoint inhibitors (ICIs). We present the case of a patient who was hospitalized with an acute alteration in mental status and was referred to the endocrinology service for evaluation of abnormal thyroid function tests. Subsequent investigation revealed the presence of acute adrenal insufficiency, which was successfully managed with steroid replacement therapy. This case highlights a unique simultaneous presentation of thyrotoxicosis due to thyroiditis and adrenal insufficiency due to hypophysitis in a patient treated with ICI avelumab for 5 months.

## Introduction

Immune checkpoint inhibitors (ICI) have become a standard therapy for various malignancies. There are currently 4 classes of ICI: cytotoxic T-lymphocyte-associated protein 4 inhibitors (CTLA-4), programmed cell death protein-1 inhibitors (PD-1), programmed cell death ligand inhibitors (PD-L1), and lymphocyte activation gene 3 inhibitors (LAG-3).

All act to overcome tumor-mediated immune inhibition, leading to a pro-inflammatory environment, which potentially increases disease control but also triggers inflammatory-mediated toxicity [1]. Consequently, there is an increased risk of developing autoimmune diseases because the T cells are no longer “held in check” and could be easily activated.

As detailed by Postow et al, potential mechanisms underlying immune-medicated side effects include increased T-cell activity against antigens both in tumors and healthy tissue, elevated levels of preexisting autoantibodies, increased inflammatory cytokine levels, and enhanced complement-mediated inflammation due to direct binding of an antibody against CTLA-4 with CTLA-4 expressed on normal tissues, such as the pituitary gland [2].

Among the immune-mediated adverse reactions, numerous endocrinopathies have been described in patients receiving ICIs, including hypophysitis, thyroid dysfunction, and primary adrenal insufficiency. Most ICI-related endocrinopathies manifest within 12 weeks after initiation of therapy, although cases have been reported to develop several months to even years after initiation [3].

Thyroid dysfunction is the most common ICI-related endocrinopathy, which may manifest as hyperthyroidism, hypothyroidism, or thyroiditis with hyper- and then hypothyroid state. Thyroid gland is known to be more susceptible to autoimmune attacks than any other organs [4]. Thyroid dysfunction occurs in up to 25% of patients treated with CTLA-4 monotherapy, 38% with PD-1/PD-L1 monotherapy, and 56% with dual CTLA-4 and PD-1/PD-L1 therapy [5].

ICI-induced hypophysitis is characterized by evidence of hypopituitarism that cannot be explained by other etiologies. A meta-analysis of 34 studies involving ICI therapy reported 85 cases of hypophysitis among 6472 patients. The incidence of hypophysitis was highest with combination therapy (6.4%), compared to 3.2% with anti-CTLA-4 therapy, 0.4% with anti-PD-1 therapy, and less than 0.1% with anti-PD-L1 therapy [6]. The hypothalamic-pituitary-adrenal axis is most often involved, followed by the hypothalamic-pituitary-thyroid axis [7].

Symptoms of endocrine dysfunction during ICI therapy can be subtle and often remain unnoticed until another hormonal axis is affected. It may unmask previously asymptomatic conditions or even make them life-threatening. We present a case of avelumab (PD-L1 inhibitor)-induced tertiary adrenal insufficiency that was accompanied by hyperthyroidism.

## Case Presentation

An 82-year-old male individual was admitted to the hospital with complaints of nausea, mild lower abdominal pain, generalized fatigue, and lethargy. The patient's medical history was significant for stage IV high-grade papillary urothelial carcinoma, diagnosed 1 year earlier, with metastasis to the liver and thoracic spine. He had undergone transurethral resection of the bladder and had been receiving chemotherapy (carboplatin and gemcitabine) for the past 9 months, and immunotherapy with avelumab for the last 5 months. Notably, he was hospitalized 2 weeks earlier for urosepsis and received antibiotic therapy, intravenous hydration, and percutaneous nephrostomy tube placement. During that admission, immunotherapy-induced pneumonitis was suspected, prompting the discontinuation of avelumab.

The patient was in moderate distress due to an altered mental status, with orientation limited to person. Physical examination revealed mild hypotension (89/60 mmHg), tachycardia (up to 105 beats per minute), dry skin, decreased skin turgor, and mild diffuse abdominal tenderness. A left nephrostomy tube was in place, draining clear urine.

## Diagnostic Assessment

Laboratory evaluation revealed elevated blood urea nitrogen of 63 mg/dL (22.5 mmol/L), (normal reference range, 6-20 mg/dL; 2.1-7.1 mmol/L), creatinine of 2.0 mg/dL (176 mmol/L), (normal reference range, 0.7-1.3 mg/dL; 44-97 mmol/L), potassium of 5.1 mEq/L (5.1 mmol/L), (normal range, 3.5 - 5.1 mEq/L; 3.5-5.1 mmol/L), sodium of 136 mEq/L (136 mmol/L), (normal range, 135-145 mEq/L; 135-145 mmol/L), phosphorus of 6.4 mg/dL (2.07 mmol/L), (normal reference range, 2.5-4.5 mg/dL; 0.84-1.45 mmol/L), and uric acid of 10.2 mg/dL (0.61 mmol/L), (normal reference range, 2.7-8.5 mg/dL; 0.24-0.51 mmol/L). No signs of infection were identified. Computed tomography (CT) of the head was negative for acute changes, and magnetic resonance imaging (MRI) of the brain revealed mild nonspecific periventricular and suprasellar T2 prolongation without apparent mass effect. Tumor lysis syndrome was suspected, and intravenous hydration was initiated. However, the patient's encephalopathy, initially attributed to metabolic derangement, persisted for 72 hours, prompting the medical team to investigate other potential causes of encephalopathy. An endocrinology consultation was requested to address abnormal thyroid function tests.

**Figure 1.**
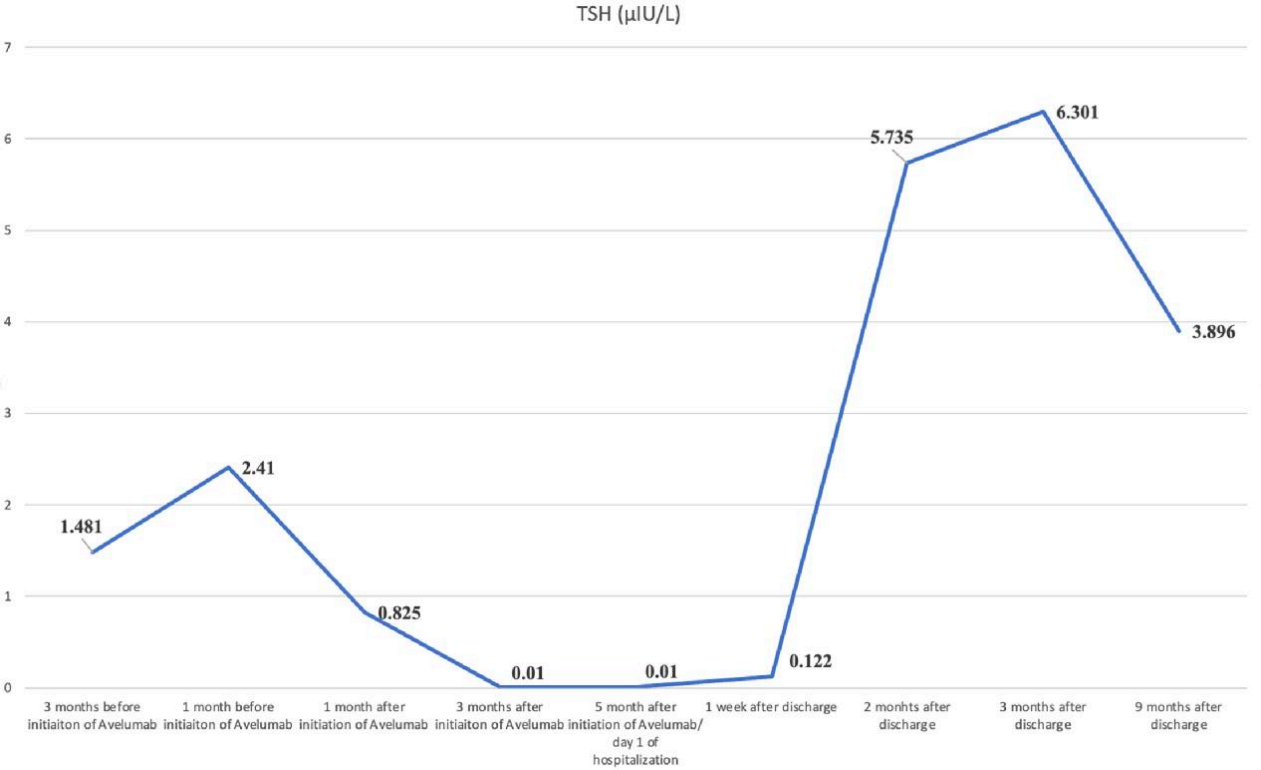
Dynamic of TSH. Dynamic of TSH before and after hospitalization. A suppressed TSH level was noted upon hospitalization, 5 months after the initiation of avelumab. This was followed by a subsequent rise in TSH, indicating a hypothyroid phase of thyroiditis, which eventually resolved, leading to the normalization of TSH levels.

Thyroid function tests indicated significantly low thyroid-stimulating hormone (TSH, <0.010 μIU/L; <0.012 ng/dL), (normal reference range 0.4–4.2 μIU/L; 0.8–1.7 ng/dL) and elevated free thyroxine (free T4, 2.28 ng/dL; 29.34 nmol/L), (normal reference range 0.9–1.7 ng/dL; 10–23 nmol/L). Notably, a decreased level of TSH was present 2 months before the presentation but was not further investigated (Fig. 1).

The endocrinology team recommended an evaluation for possible adrenal insufficiency. A morning cortisol level of 0.57 mcg/dL (15.73 nmol/L), (normal reference range, 5-23 mcg/dL; 138-635 nmol/L) was obtained, followed by a repeat test showing 0.67 mcg/dL (18.49 nmol/L), with an adrenocorticotropic hormone (ACTH) level measured at 1.5 pg/mL (0.33 pmol/L), (normal reference range, 9-52 pg/mL; 2-11 pmol/L). Additional pituitary hormone levels were checked, revealing normal levels of follicle-stimulating hormone (FSH), luteinizing hormone (LH), prolactin, and insulin-like growth factor-1 (IGF-1).

A thyroid ultrasound showed a small, heterogeneous thyroid gland with no discrete nodules and decreased blood flow. Thyroglobulin, thyroid peroxidase, and thyrotropin receptor antibodies were normal.

## Treatment

Hydrocortisone therapy was initiated at a dose of 50 mg every 8 hours, which was later titrated down to 10 mg in the morning and 5 mg in the evening. Given the clinical signs suggestive of hyperthyroidism, including mild tachycardia and altered mental status, alongside elevated thyroid hormone levels, a decision was made to start low-dose methimazole at 5 mg once daily and metoprolol 75 mg daily.

## Outcome and Follow-Up

Over the following week, the patient's mental status significantly improved and returned to baseline by the time of discharge. Follow-up thyroid function tests showed a significant steady decline in free T4 levels, prompting a reduction in the methimazole dose to 2.5 mg daily and subsequent discontinuation after the discharge. The patient was discharged on the twelfth day of hospitalization. Hydrocortisone therapy was continued after discharge in the dose of 10 mg in the morning and 5 mg in the evening. At follow-up 3 months later, the patient was asymptomatic, and his laboratory parameters had normalized. He has been remaining euthyroid off methimazole for over a year following discharge, indicating resolution of thyroiditis.

## Discussion

As the list of indications for ICIs continues to extend, so does the range of their side effects, including endocrinopathies. We report a case where 2 different endocrinological disorders, hypophysitis and thyroiditis, co-presented. These disorders had overlapping manifestations and could have been life-threatening if not promptly recognized.

There are only a few brief abstracts and case reports describing the simultaneous involvement of multiple endocrinological systems following ICI therapy [8-11]. Most of these cases are associated with combination therapy or the PD-1 class of ICI. This is the first case of ICI-induced multiple endocrinopathies following the administration of PD-L1 inhibitor avelumab, presenting with simultaneously diagnosed thyrotoxicosis due to thyroiditis and adrenal insufficiency due to hypophysitis.

**Figure 2.**
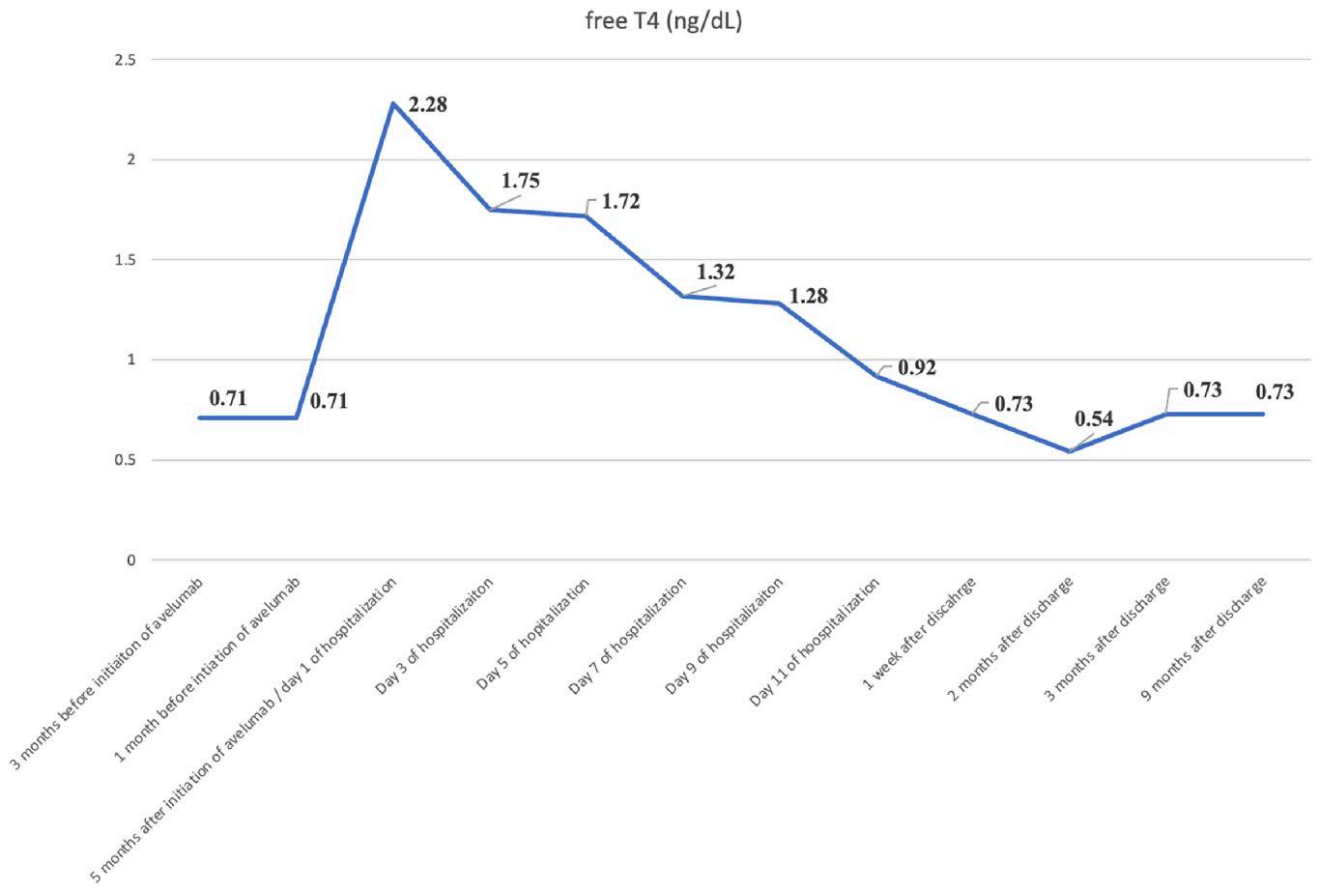
Dynamic of free T4. Dynamic of free T4 before and after hospitalization. The hyperthyroid state transitioned into a transient hypothyroid state 1 week after hospitalization. Eventually, thyroid function tests returned to baseline with the resolution of thyroiditis.

Low TSH levels recorded 3 months after initiating PD-L1 inhibitor avelumab (Fig. 1) indicated the presence of a hyperthyroid state. However, further endocrine investigations were not conducted at that time. Hyperthyroidism (Fig. 1, Fig. 2) was diagnosed during hospitalization. The negative thyroid antibody profile, reduced thyroid blood flow on Doppler ultrasound, and rapid progression from hyperthyroidism to euthyroidism were highly suggestive of thyroiditis as the underlying cause of the transient thyrotoxicosis. Although we suspected thyroiditis, we opted to administer a low dose of methimazole initially to facilitate the rapid resolution of transient hyperthyroidism. This approach was particularly suitable as we were able to closely monitor the patient and promptly reduce and discontinue the medication once euthyroid state was achieved.

The diagnosis of hypophysitis became evident when central adrenal insufficiency was confirmed.

The nonspecific nature of ICI-induced hypophysitis symptoms may complicate timely diagnosis. In our case, the main manifestation of adrenal insufficiency was an acute change of mental status, while hemodynamic parameters showed only intermittent hypotension. However, acute change of mentation is considered one of the typical signs of adrenal crisis. Neurological symptoms of adrenal crisis have been an area of interest for clinicians for a long time. Glucocorticoid receptors are abundant in the hippocampus and other areas of the brain. It was ultimately concluded that suppression of endogenous glucocorticoids was sufficient to produce prefrontal cortex dysfunction and thus provided evidence that glucocorticoids are essential for cognitive function [12]. Psychosis and extensive cognitive changes, including delirium, appear to occur more rarely, but are associated with severe disease and may be the presenting feature of Addisonian crisis [13]. The rapid improvement in mental status, stabilization of hemodynamics, and correction of electrolyte abnormalities following steroid replacement, supports adrenal insufficiency as the primary driver of clinical deterioration.

The fact that the patient was hospitalized for urinary tract infection 2 weeks prior to the current admission, with normal hemodynamic parameters and cognition, points to the acute onset of adrenal insufficiency. On the other hand, the presentation of adrenal insufficiency could also be unmasked in the setting of overt hyperthyroidism. In thyrotoxicosis there is an increased need for stress hormones due to increased metabolic demands which is compounded by the fact that thyroid hormones increase hepatic corticosteroid metabolism, thereby precipitating an Addisonian crisis.

Numerous studies have shown that the risk of endocrinopathy due to ICI use is significantly higher when multiple organ systems are involved. In our case, the pulmonology team was also concerned about ICI-induced pneumonitis.

According to the American Society of Clinical Oncology (ASCO) Guidelines [14], thyroid function tests should be screened every 4 to 6 weeks following the initiation of ICI therapy, or sooner in symptomatic patients. However, screening for pituitary dysfunction is not routinely performed and is generally based on the presence of symptoms. The Society for Immunotherapy of Cancer (SITC) Toxicity Management guidelines recommend checking baseline morning cortisol and ACTH levels before starting treatment, followed by monthly monitoring for 6 months, then every 3 months for 6 months, and every 6 months for 1 year thereafter [15].

In conclusion, a favorable outcome was achieved with the complete resolution of the patient's symptoms and a return to baseline physical and mental function. However, considering the severity of the condition and its potential for significant morbidity and mortality, a proactive approach to early detection of endocrinopathies is crucial in the management of patients receiving ICI.

## Learning Points

- Immune checkpoint inhibitors (ICIs) are now widely used in the treatment of various cancer types. Numerous endocrinopathies have been reported in patients receiving ICIs, including hypophysitis, thyroid dysfunctions, and primary adrenal insufficiency.
- Symptoms of endocrine dysfunction can be subtle and often go unnoticed until another hormonal axis becomes involved. In our patient, thyrotoxicosis contributed to the manifestation of adrenal crisis.
- Periodic thyroid function tests and hypothalamic-pituitary-adrenal axis screening are recommended for patients after starting ICI therapy or as needed in symptomatic cases. Early recognition of endocrinopathies is essential to prevent significant morbidity in affected patients.

## Contributors

All authors made individual contributions to authorship. All authors were involved in the diagnosis, management, and follow-up of the patient. Z.Z. and M.K. were involved in writing the manuscript and preparing the images. H.G. was engaged in the final edition of the text. All authors reviewed and approved the final draft.

## Data Availability

Original data generated and analyzed during this study are included in this published article.

## Abbreviations

ACTH: adrenocorticotropic hormone
CTLA-4: cytotoxic T-lymphocyte-associated protein 4
ICI: immune checkpoint inhibitor
PD-1: programmed cell death protein-1
PD-L1: programmed cell death ligand
T4: thyroxine
TSH: thyrotropin (thyroid-stimulating hormone)
